# Diabetes Is a Community Issue: The Critical Elements of a Successful Outreach and Education Model on the U.S.-Mexico Border^1^

**Published:** 2004-12-15

**Authors:** Maia Ingram, Gwen Gallegos, JoJean Elenes

**Affiliations:** Mel and Enid Zuckerman Arizona College of Public Health; Carondelet Health Network, Holy Cross Hospital, Nogales, Ariz; Mariposa Community Health Center, Nogales, Ariz

## Abstract

**Background:**

Diabetes is reaching epidemic proportions on the U.S.-Mexico Border, and culturally competent diabetes education is not available in many communities.

**Context:**

People with diabetes often do not have access to regular medical care, cannot afford medication, and lack the community infrastructure that supports self-management practices. Self-management education and support have great potential to impact diabetes control in this environment.

**Methods:**

To address this need, partners of the Border Health Strategic Initiative *(Border Health ¡SI!)* collaboratively developed a culturally relevant diabetes outreach and education program. The model included a five-week series of free diabetes education classes that assisted participants in gaining the knowledge and skills necessary to be physically active, control diet, monitor blood sugar, take medications, and be aware of complications. Central to the model was the use of community health workers — or *promotores de salud* — to conduct outreach, participate in patient education, and provide individual support.

**Consequences:**

Program participants achieved significant improvements in self-management behaviors and HbA1c, random blood glucose, and blood pressure levels.

**Interpretation:**

Quantitative and qualitative evaluation helped to identify the essential elements of a successful program, including partnership of providers, community diabetes classes, *promotores* outreach and support, linkage between diabetes education and clinical care, and program evaluation.

## Background

The impact of diabetes is devastating along the U.S.-Mexico Border. The rate of diabetes mortality in the border region is nearly 50% higher than in the rest of the country (1), and Hispanics are two to three times more likely to suffer from serious secondary complications (2,3). Self-management behaviors, such as diet, physical activity, and glucose self-monitoring are fundamental to avoiding the long-term complications of diabetes (4). For many individuals, however, self-management behaviors constitute drastic lifestyle changes for which there is little external support. In a managed-care setting, Hispanics were shown to exhibit poor diabetes control when compared with non-Hispanic whites (5).

Diabetes education can have a positive impact on self-management behaviors and glycemic control, particularly when accompanied by intensive follow-up support (6). Diabetes classes delivered in a community setting have been shown to be effective in achieving glycemic control among adults with type 2 diabetes, and this mode of delivery is likely to increase the cultural relevancy and appropriateness of educational techniques in addition to providing greater access to hard-to-reach populations (7). Community partnerships also have the potential to enhance cultural relevance and positively impact self-management and clinical outcomes (8).

There are overwhelming challenges to providing formal diabetes education in border communities. Individuals without insurance do not have access to diabetes education services. For individuals with insurance, few certified diabetes educators (CDEs) live and work in border communities and they may not speak Spanish. Programs that provide interpretation or translation are often not culturally relevant to Hispanics.

This paper describes the patient component of the Border Health Strategic Initiative (*Border Health ¡SI!*) funded by the Centers for Disease Control and Prevention (CDC), which used the community health worker model to provide culturally competent diabetes education in two Arizona border communities in Yuma and Santa Cruz counties. A detailed description of *Border Health ¡SI!* is included in this issue of *Preventing Chronic Disease* (9) along with several companion papers addressing other components of the model (10-18). More information on the rationale and effectiveness of the community health worker model in addressing diabetes can be found in the CDC Division of Diabetes Translation's position statement (available from http://www.cdc.gov/diabetes/projects/comm.htm).

## Context

Individual ability to manage diabetes cannot be separated from community context and support for diabetes care (19). Both Yuma and Santa Cruz Counties are rural and more than 90% Hispanic; Yuma County has a large migrant/farmworker community. The region is medically underserved. Lack of insurance, seasonal employment of farmworkers, and fear and discrimination related to immigration present challenges to establishing a regular source of care (20). Patients with diabetes often cross the border to Mexico for medical care, making it difficult to maintain continuity of care.

Residents not eligible for Medicaid programs can rarely afford diabetes medication. Individuals with insurance often do not have pharmaceutical coverage and must decide whether to buy food or medicine. Patients share medication or resort to taking it only when they are feeling badly. While diabetes programs may make glucose monitors available, few resources cover the cost of glucose-monitoring strips.

The border environment does not support good nutrition and physical activity. Few recreational areas, parks, or sidewalks exist in these rural areas to facilitate walking. Summer heat, inadequate lighting, dangerous walking surfaces, and wild dogs pose additional challenges. Although southern Yuma County is a farming community, and the city of Nogales (in Santa Cruz County) is a throughway for produce from Mexico, healthy foods such as fresh fruits and vegetables are high-priced and often unavailable. Furthermore, the health messages taken for granted in urban areas rarely reach farmworkers who work 12-hour days in isolated areas.

The social network that can potentially support self-management is often not in place. The elderly may have family members who migrate to follow the harvesting season or move to urban areas. Many extended family members live in Mexico. Diabetes patients may become isolated and depressed as they experience increasing health problems.

Because of these barriers, education programs must be culturally competent. Vital to the diabetes education program was the use of *promotores de salud. Promotores* are indigenous to the communities in which they work and provide a bridge between the health care delivery system and the community. In addition to health information, they provide social support and advocate for patients to gain access to health and social services (21). In one diabetes education program, the use of *promotores* in a Hispanic community was shown to increase the rate of completion (22).

### The program

The diabetes outreach and education program was created in Santa Cruz County under a Health Resources and Services Administration Rural Health Outreach Grant (RHOG) in 1997 and adapted by the Yuma community in 2000 under its own RHOG. The programs were supported logistically under the comprehensive framework of *Border Health ¡SI!* over a three-year period, although Yuma County had additional resources. An investigation of both programs allowed us to define the essential elements of the outreach and education model, which are described below and illustrated conceptually (Figure).**

*Partnership of providers.* Both the Yuma and Santa Cruz programs relied upon a consortium of community providers to implement the patient education component. The community health centers (CHCs) administered the programs and provided a program coordinator. Both programs involved first-time collaboration between the health center and local hospital. The hospital in each county provided a CDE to facilitate classes, train *promotoras* in diabetes care, and work individually with participants. In Yuma, a grassroots farmworker advocacy organization provided the *promotoras*, while in Santa Cruz, the *promotoras* were provided by the CHC. Each program had an academic partner who provided evaluation and technical assistance. The collaborative aspect of the program was crucial in building broader community support for diabetes care.

**Figure 1:**
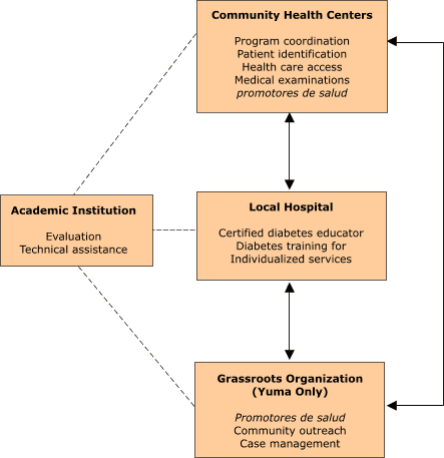
The roles and responsibilities of partners in the diabetes outreach and education program, *Border Health ¡SI!*, Yuma and Santa Cruz counties, Arizona. *Promotores de salud* are community health workers.

*Community diabetes classes*. Very few participants had prior diabetes education, although many had had diabetes for years. The programs used a culturally competent curriculum that employed a variety of teaching methods to educate participants on how diabetes affects the body and how self-management controls the disease. The curriculum was developed prior to the initiation of the programs by the CDE working in Santa Cruz County using the American Diabetes Association (ADA) Standards of Care. The curriculum followed the content areas set by the ADA and adapted them to the border communities. The curriculum included five two-hour classes held once a week over a five-week period. The sessions included the following topics: 1) understanding diabetes; 2) meal planning; 3) monitoring, medications, and movement; 4) avoiding complications and maintaining health; and 5) foot/eye clinics. In both sites, participants were encouraged to bring family members. The class formats included presentation and discussion and used handouts, videos, and other teaching aids, such as food models. Participants engaged in activities such as creating a balanced plate of food to achieve dietary goals and dancing to achieve physical activity goals. Each class began with a review of the previous session. In addition, program staff measured blood glucose, weight, and blood pressure at each class to demonstrate to participants the progress they were making over the course of the program.

In Santa Cruz, a bicultural CDE based in the local hospital taught the classes. In Yuma, classes were taught by a health educator and eventually by the *promotoras* under the supervision of a non-Spanish–speaking CDE located in the hospital. Class structure varied between communities. The Santa Cruz community embraced the importance of an open-door program so that classes were available on a rotating basis and class size was maintained at about 20 participants. Participants attended them in any sequence and as often as they wished. In Yuma, the partners recruited a group for each round of classes and encouraged them to complete the program during this time period. Growing interest in the Yuma program resulted in class sizes of up to 40 people.

*Promotores outreach and support.* The role of the *promotoras* was to provide outreach, assist participants in incorporating self-management behaviors into their lifestyles, and offer ongoing support and follow-up. There was some disparity in resources between the two programs, and the Yuma community had the advantage of being able to fully implement the *promotora* model. Four *promotoras* took responsibility for recruitment, support, and follow-up for a caseload of participants. Potential participants were identified through the health center database. The *promotoras* personally invited potential participants to the program, provided support to the learning process both during and outside of the classes, and followed up with participants for a six-month period following completion of the classes. The *promotoras* assisted patients in accessing health insurance, medications, and other social services.

In Santa Cruz, one *promotora* was available on the day of class to provide telephone follow-up. However, because this program had been initiated several years prior to the initiation of the *Border Health ¡SI!* patient education component, local providers were aware of the benefits of the program and regularly referred their patients to the classes.

*Linkage between diabetes education and clinical care*. In both communities, the program was based in a CHC, increasing opportunities for patient-provider communication on patient care. During the program, providers in both programs increased referrals as they recognized the benefits of patient participation. In Santa Cruz, the program added a patient diabetes "empowerment card" to track clinical care and increase patient-provider communication. The trifold card included a form to track the last five physician visits, current medications, participation in diabetes classes, and personal goals.

Many participants did not have access to regular care, and many could not recall a past eye examination. The programs assisted participants in identifying insurance options. A foot exam was included in both programs, and in Yuma, ophthalmologists volunteered their time for eye clinics on Saturdays.

Regardless of insurance status, many participants could not afford medication. While program resources to provide medication were not available, participants were linked to insurance or special programs when possible. Both programs accessed samples from pharmaceutical companies.

*Program Evaluation.* Program partners engaged in a participatory model of evaluation under the guidance of the academic institution. Under the participatory model, all stakeholders are involved in each phase of evaluation, ensuring a continuous exchange of knowledge, skills, and resources (23). Partners collaboratively developed quantitative and qualitative instruments and shared responsibility for data collection. The academic partner was responsible for analyzing and compiling program data on a cyclical basis to allow for integration of program findings over time. Evaluation efforts were hindered, however, by a lack of resources, which resulted in gaps in data and at times forced *promotoras* to choose between serving clients (always the first priority) and collecting evaluation information.

Self-management practices were assessed through pre- and follow-up questionnaires administered by the *promotoras* prior to initiation in the program and six months after graduation. The academic partners trained *promotoras* in administering the questionnaire, which asked participants if they engaged in self-management practices, including diet, physical activity, foot care, and regular glucose monitoring. The questionnaire also asked participants about their most recent visit with their doctor and whether they had received diabetes health exams in the past year. The initial questionnaire included information on demographics and health history.

Health outcomes included random blood glucose, blood pressure, weight, and HbA1c. Program staff took measurements at three points: initiation of classes, upon graduation from the program, and six months afterwards. HbA1c was measured only twice: before classes and at six-month follow up. In Yuma, the data set is much more complete than in Santa Cruz, and all post-measures were made six to 12 weeks after participants entered the program. In Santa Cruz, the timing of post-measures varied because participants graduated at different points, and attempts to collect HbA1c data at follow-up were unsuccessful because of a lack of staff and financial resources.

Qualitative evaluation took place in Yuma and consisted of in-depth interviews with a random sample of participants in the second and third years of the program. Program partners developed the questionnaire, and academic partners who were not engaged in service delivery conducted the interviews. The interviews explored perceptions of diabetes before and after the program, the role of the family in self-management, changes in self-management practices, and ongoing barriers to diabetes control.

## Consequences

The process of implementing the *Border Health ¡SI!* patient education component over three years in two communities provided a rich opportunity to learn from successes and challenges. In spite of diminishing resources, both programs maintained a strong commitment to providing diabetes education to the underserved. Both communities expressed increased demand for the classes, which was difficult to manage in Yuma because the program moved one group of participants through one series of classes before starting another. At times, classes in Yuma had more than 40 people. Santa Cruz began offering classes in the evening to respond to those who worked during the day.

Santa Cruz had the advantage of a CDE who had worked in the community for years. The Yuma health educator left halfway through the program. The *promotores* then took responsibility for teaching the classes under the supervision of the hospital CDE. Participant outcomes were maintained when the *promotores* began teaching.

### Evaluation results

Evaluation results generated by the *Border Health ¡SI!* patient education component are extensive; this paper attempts only to highlight key findings. Table 1 describes the characteristics of individuals who enrolled in the diabetes education classes. In Yuma, 376 individuals enrolled in classes and 306 (81%) graduated. Of graduates, 243 (79%) were reached for the follow-up interview. In Santa Cruz, 406 people enrolled in classes, and 135 (33%) graduated. Of graduates, 40 (30%) were reached for follow-up. Demographic information revealed that the programs did reach the targeted populations. In both counties, participants were more likely to be female and older than 50 years. The majority did not graduate from high school, and approximately two thirds had family members with diabetes. In Yuma, participants were slightly older and experienced more diabetes-related illness; however, they had better access to insurance through Medicare. Few participants had received prior diabetes education, and many had never had an eye exam. Approximately one half reported having high blood pressure and, in Yuma, 59% experienced numbness and burning in their feet.

#### Health outcomes

Health measures were taken pre- and post-class and at six-month follow-up. Paired *t*-tests performed on pre- and post-data revealed a significant decrease in the average random blood glucose measurement among participants in both programs (Table 2). In Yuma, levels dropped from 224 mg/dL to 201 mg/dL, and, in Santa Cruz, levels dropped from 197 mg/dL to 151 mg/dL. Both programs also achieved modest but significant decreases in diastolic blood pressure among all participants. Among high-risk participants in Yuma, systolic blood pressure fell from 151 mg/dL to 137 mg/dL, and diastolic blood pressure fell from 100 mg/dL to 84 mg/dL. Among-high risk participants in Santa Cruz, systolic blood pressure fell from 153 mg/dL to 139 mg/dL, and diastolic blood pressure fell from 102 mg/dL to 91 mg/dL. There were no significant changes in health outcomes at the six-month follow-up measure. In Yuma, follow-up results demonstrated a significant 0.7 decrease in HbA1c from 9.4 to 8.7 among those who initiated the program with HbA1c >6.9.

#### Self-management outcomes

Self-management practices were evaluated in the six-month follow-up interview. Paired *t*-tests were used to determine significant changes in self-management behaviors. As seen in Table 3, a significant proportion of participants in both counties reported increasing self-management behaviors, including diet, foot care, and glucose monitoring. In Santa Cruz, the percentage of individuals following a diabetes diet increased significantly. In Yuma, where HbA1c and eye exams were provided as part of the *Border Health ¡SI!* patient education component, the percentage of individuals who had ever received these examinations increased significantly from 53% to 96% (HbA1c) and 57% to 91% (eye exam).

#### In-depth interviews

Quality of life is as important as clinical outcomes, and in-depth interviews in Yuma demonstrated the impact of the program on program participants. Participant attitude toward diabetes changed from ignorance and fear to acceptance and control, which seemed pivotal in improving their emotional well-being, regardless of self-management practices. Comments included:

- "I take care of myself better. I know what is bad for me. I don't feel angry now."
- "They tell you how to care for yourself. You can adapt and live a normal life."

The *promotoras* were also vital to the process because participants felt that the *promotoras* cared for them and were willing to do whatever they could to help them.

- "They are concerned about me. I am motivated because they are worried about me and helped me. "
- "My *promotora* is marvelous. I have a thousand good things to say about her."

Both programs used findings to pursue and secure additional funding to sustain services.

## Interpretation

This program responded to a need for accessible, culturally competent diabetes education and demonstrated how communities can galvanize local capacity to respond to an overwhelming lack of resources. Local providers contributed free eye and foot exams and *promotoras* took over the diabetes education classes when the health educator left the community.

*Partnership of providers*. Crucial to success was the partnership of diverse organizations that enabled the programs to confront challenges of the border environment on multiple levels. The CHCs had access to the target population, but they would not have been able to recruit and retain participants without the *promotores*. In both communities, the hospital was critical in providing expertise and in accessing resources.

*Community diabetes classes*. Holding classes at a community site in a series with a specific group of participants appears to contribute to program completion. This may be because participants have a greater sense of commitment and enjoy belonging to a group. Santa Cruz was extremely fortunate to have a committed, culturally competent and expert CDE. In rural communities where CDEs are not available, *promotoras* can be trained to provide diabetes education. It is vital, however, that they have backup and support from a qualified person.

*Promotores outreach and support.* Program outcomes would not have been achieved without *promotores*. *Promotores* are fundamental in ensuring that participants initiate and complete classes, gain access to resources, and adopt self-management practices.

*Linkage between diabetes education and clinical care*. Providing access to health care, examinations, and medications is a challenge that should be addressed early on. For this reason alone, community collaboration is essential. Creating formal relationships with clinical providers may enhance health outcomes. The patient empowerment card was one attempt to establish a formal relationship, and the card was popular with program participants. Strategies to ensure that providers use the card need to be implemented and the impact on care needs to be evaluated.

*Program evaluation.* Conducting meaningful program evaluation — especially with limited resources — was a challenging but key element of the patient education component. Consistent with the participatory model of evaluation, the academic partner was not an outsider to but rather an integral member of the team and a stakeholder in its success. Within this framework, evaluation became a tool of program development, encouraging partners to define concretely the desired outcomes of the program, to make the effort to collect the necessary information, and to integrate feedback into program strategies. The influence of evaluation on *Border Health ¡SI!* included 1) designing a series of diabetes education classes (rather than an open-door policy) to create group cohesion and support, 2) establishing a greater focus on including family members in the education and care process, and 3) developing strategies to increase patient-provider communication. Both *Border Health ¡SI!* communities used evaluation results to sustain program activities beyond the funding period, one through institutional support and the other through other grant funding.

In these two marginalized border communities, the *Border Health ¡SI!* diabetes education and outreach program had a positive influence on the ability of individuals to adopt self-management practices and improve health outcomes. It is important to note that as a component of the comprehensive *Border Health ¡SI!*, the education and outreach program was linked to a policy action group that addressed challenging environmental issues related to diabetes (15,16). Participation in a policy-focused group enabled program partners and community leaders to discuss systemic problems, leverage additional resources, and address prevention on a community level.

## Figures and Tables

**Table 1:** Characteristics of Participants in Diabetes Patient Education Program, Border Health Strategic Initiative, Arizona, 1999–2002

	***Santa Cruz County*** N = 406 ***(%)***	***Yuma County*** N = 376 ***(%)***
**Female**	284 (70)	250 (66)
**Aged >50 years**	203 (50)	262 (70)
**Graduated from high school**	170 (42)	72 (19)
**Insured**	268 (66)	281 (75)
**Diabetes in family**	276 (68)	275 (73)
**HbA1c >6.9**	Data not available	212^a^ (58)
**Prior diabetes education**	28 (7)	64 (17)
**High blood pressure**	191 (47)	196 (52)
**Numbness/burning in feet**	138 (34)	218 (59)
**Hospitalized in the last year for diabetes**	46 (11)	67 (18)
**Graduated from program**	135 (33)	306 (81)

a N = 290 because of missing data.

**Table 2:** Changes in Health Measurements Among Participants Who Completed Diabetes Patient Education Program, Border Health Strategic Initiative, Arizona, 1999–2002^a^

	***Santa Cruz County*** ***N = 135***		***Yuma County*** ***N = 306***	

**Among all participants**				

	**Pre-program**	**Post-program**	**Pre-program**	**Post-program**
**HbA1c level (N = 198)**	No data available		8.7	8.2**
**Random blood glucose (mg/dL)**	196.9	151.1***	224.5	200.6***
**Systolic blood pressure (mm Hg)**	130.3	128.2	131.5	127.9***
**Diastolic blood pressure (mm Hg)**	80.9	78.0*	77.9	76.5*
**Weight (lbs)**	184.6	182.7*	174.3	173.0*

**Among high-risk participants**				

**HbA1c level (N = 132)**	No data available		9.4	8.7***
**Random blood glucose (mg/dL)**	225.9	159.6***	246.6	212.0***
**Systolic blood pressure (mm Hg)**	152.6	138.8***	150.8	137.3***
**Diastolic blood pressure (mm Hg)**	101.6	91.3***	99.6	84.5***

a Post-program measurements were taken upon completion of the program, with the exception of HbA1c, which was taken at six-month follow-up. High-risk is defined as HbA1c level >6.9. P = *<.05; **<.01; ***<.001.

**Table 3:** Changes in Self-Reported Diabetes Self-Management Outcomes, Border Health Strategic Initiative, Arizona, 1999–2002^a^

	***Santa Cruz County*** ***N = 40***		***Yuma County*** ***N = 243***	

	**Pre-program**	**Post-program**	**Pre-program**	**Post-program**
**Exercises regularly**	50	70*	67	83***
**Follows diet**	45***	80***	Data not complete	
**Checks feet regularly**	60	88**	86	98***
**Monitors blood sugar**	38	63	51	96***
**Ever had HbA1c**	33	45	53	96***
**Knows what HbA1c is**	40*	40*	22	64***
**Ever had eye exam**	33	47	57	91*

a All values are percentages. Post-program measurements were taken six months after program graduation. P = *<.05; **<.01; ***<.001.
